# Exploratory prescribing risk profiles and drug drug interaction burden in older adults with depressive episodes

**DOI:** 10.3389/fpsyt.2026.1915707

**Published:** 2026-08-21

**Authors:** Florina-Diana Goldiş, Sebastian-Mihai Ardelean, Lucreţia Udrescu

**Affiliations:** 1 Center for Drug Data Analysis, Cheminformatics, and the Internet of Medical Things, Victor Babeş University of Medicine and Pharmacy, Timişoara, Romania; 2 Doctoral School of Pharmacy, Victor Babeş University of Medicine and Pharmacy, Timişoara, Romania; 3 Department of Computer and Information Technology, Politehnica University, Timişoara, Romania; 4 Department of Clinical Pharmacy and Drug Analysis, Victor Babeş University of Medicine and Pharmacy, Timişoara, Romania

**Keywords:** drug-drug interactions, late-life depression, medication safety, polypharmacy, prescribing risk profiles, psychogeriatrics, psychopharmacotherapy

## Abstract

**Introduction:**

Older adults with depressive episodes are frequently treated in the context of psychiatric multimorbidity, somatic co-treatment, and polypharmacy, increasing their vulnerability to drug-drug interactions (DDIs) and cumulative medication-related risk. This study characterized DDI burden and exploratory prescribing risk profiles among psychogeriatric outpatients, focusing on patients with depressive episodes.

**Methods:**

We conducted a retrospective observational study of psychiatric outpatient visits in 2023. The primary subgroup consisted of those diagnosed with a depressive episode. Patients with at least one diagnosis of a depressive episode formed the primary depression-focused subgroup. Potential DDIs were assessed using DrugBank and classified as major, moderate, or minor. These potential DDIs were database-identified and were not clinically confirmed adverse drug events. Visit-and patient-level analyses described potential DDI burden, medication risk burden, psychiatric co-diagnoses, and diagnosis-specific potential DDI pairs. Multivariable logistic and negative binomial regression models were fitted among patients with depressive episodes. Patient-level exploratory prescribing risk profiles were derived in the full cohort using DDI and medication risk burden features.

**Results:**

The cohort included 309 patients and 1242 eligible visits; 242 patients had at least one depressive episode diagnosis. Anxiety disorders were the predominant co-diagnosis among patients with depressive episodes, being recorded in 193 patients (79.8%) and co-occurring with depressive episode at the same visit in 177 patients (73.1%). Nearly half (50.4%) of visits from depressive-episode patients included at least one database-identified major potential DDI, while 93.7% had at least one moderate potential DDI. Leading moderate DDI pairs mainly involved benzodiazepine/Z-drug and benzodiazepine/antidepressant combinations. Leading major DDI pairs included cardiovascular, carbamazepine-related, and trazodone-related interactions. Medication load was the variable most consistently associated with potential DDI burden, while diagnostic complexity contributed to cumulative annual major DDI burden. A higher-burden exploratory prescribing risk profile identified 86 patients with depressive episodes (35.5%), highlighting a clinically important subgroup characterized by greater polypharmacy, potential DDI burden, and geriatric medication risk scores.

**Conclusion:**

Database-identified potential DDI burden was substantial among older adults with depressive episodes and was driven mainly by medication load. Anxiety overlap, recurrent sedative-related moderate potential DDIs, and higher-burden prescribing risk profiles highlight the need for structured, regimen-level medication review.

## 1 Introduction

Polypharmacy in older adults is complex because it occurs in the context of age-related physiological change, multimorbidity, and exposure to multiple concomitant drugs. Altered pharmacokinetic parameters and central nervous system sensitivity may modify drug response in later life, increasing susceptibility to adverse drug events and reducing the margin of safety for psychiatric prescribing Kratz and Diefenbacher (1)Jacobson (2)Błeszyńska et al. (3). In this context, potentially inappropriate medications (PIMs) and drug-drug interactions (DDIs) become especially important because they can worsen tolerability, reduce adherence, impair quality of life, and contribute to treatment failure Moebs et al. (4)O’Mahony et al. (5)Pazan and Wehling (6).

Multimorbidity, usually defined as the coexistence of at least two chronic conditions, is common in later life and often necessitates long-term pharmacological treatment Skou et al. (7). As a result, polypharmacy is highly prevalent in older adults and even more frequent in psychiatric populations, where recurrent episodes, symptom clustering, and somatic comorbidity often lead to layered prescribing Pazan and Wehling (6)Toh et al. (8)Moreira et al. (9)Al Zaabi et al. (10). At the same time, not all polypharmacy is inherently inappropriate. In affective, psychotic, and bipolar disorders, combination treatment may be clinically justified, but the distinction between rational and irrational polypharmacy is crucial. Irrational combinations, such as drugs from the same class with overlapping pharmacodynamic effects or drugs prone to clinically meaningful pharmacokinetic interactions, can amplify toxicity without adding therapeutic benefit Zigman and Blier (11)Preskorn and Lacey (12)Sarkar (13). An alternative perspective is appropriate polypharmacy, focusing on optimizing treatment and deprescribing excess treatment instead of merely counting drugs O’Donnell and Ibrahim (14)Linsky et al. (15).

DDIs may be pharmacokinetic or pharmacodynamic. Pharmacokinetic DDIs alter absorption, distribution, metabolism, or elimination, often through cytochrome P450 inhibition or induction, and are especially relevant in older adults because hepatic and renal reserve decline with age U.S. Food and Drug Administration (16)Hefner et al. (17)Tulner et al. (18). Pharmacodynamic DDIs reflect additive or synergistic effects at the level of sedation, orthostatic hypotension, anticholinergic load, QT prolongation, electrolyte disturbance, or bleeding risk. These mechanisms are clinically meaningful in geriatric psychiatry, where the same patient may receive psychotropics, cardiovascular drugs, cognitive enhancers, analgesics, and gastrointestinal drugs. Previous studies indicate that clinically important DDIs are common in older populations and may contribute to hospital admission, bleeding, falls, prolonged hospitalization, and reduced quality of life Tulner et al. (18)Hughes et al. (19).

Older psychiatric patients may face multiple overlapping drug-related risks. Anticholinergic burden is associated with impaired attention, memory, and cognition, particularly in patients with pre-existing neuropsychiatric vulnerability Boustani et al. (20)Collamati et al. (21)López-Álvarez et al. (22)Taylor-Rowan et al. (23)Peralta et al. (24). Psychotropics may also increase QT-related arrhythmic risk, especially when combined with other QT-prolonging drugs or used in multimorbid older adults with structural or autonomic cardiac vulnerability Beach et al. (25)Meid et al. (26)Ramasubbu et al. (27)Woosley et al. (28). Additionally, several antidepressants, antipsychotics, antiepileptics, and diuretics may contribute to syndrome of inappropriate antidiuretic hormone secretion (SIADH) or hyponatremia, an under-recognized geriatric syndrome that can mimic or worsen cognitive impairment Cuesta and Thompson (29)Warren et al. (30). PIM frameworks such as the Beers criteria, STOPP/START, and PRISCUS were developed specifically because older patients are vulnerable not only to single high-risk drugs, but also to cumulative prescribing hazards across classes and mechanisms O’Mahony et al. (5)American Geriatrics Society (31)Thürmann et al. (32).

Although the medication-safety challenges of geriatric psychopharmacology are well recognized, less is known about how potential DDI burden accumulates across visits and patients with late-life depressive episodes in routine outpatient care. This gap is clinically relevant because depression in older adults commonly coexists with anxiety, insomnia, cognitive impairment, vascular disease, and multimorbidity, leading to treatment regimens that combine antidepressants, anxiolytics, hypnotics, antipsychotics, cognitive enhancers, cardiovascular drugs, and other somatic medications Devita et al. (33)Hu et al. (34)Kok and Reynolds III (35)Read et al. (36). Previous studies have linked polypharmacy and DDIs in older adults to adverse drug events and other clinically relevant outcomes, but fewer studies have jointly examined visit-level and patient-level potential DDI burden, diagnosis-specific interaction patterns, and cumulative geriatric medication-risk domains such as anticholinergic burden, QT prolongation/torsades de pointes (QT/TdP) risk exposure, Beers criteria exposure, and SIADH risk in psychogeriatric outpatient depression Tulner et al. (18)Hughes et al. (19)Maher et al. (37)Rankin et al. (38)Davies and O’mahony (39)Onder et al. (40). Recent outpatient pharmacoepidemiologic studies further support the relevance of this question. Large-scale prescription data analyses have shown that antidepressant prescribing is common and varies by age, sex, and antidepressant class, with selective serotonin reuptake inhibitors often predominating in outpatient practice Karami et al. (41). In older outpatients, studies based on the Beers criteria also show a substantial burden of PIMs and polypharmacy, reinforcing the need for structured medication safety assessment in geriatric prescribing Gholamnezhad et al. (42). In parallel, national outpatient DDI analyses indicate that potential DDI exposure is strongly related to polypharmacy and specific prescribing patterns, including psychiatry as a high-risk prescribing context Aarabi et al. (43).

Accordingly, we aimed to characterize database-identified potential drug-drug interaction burden and medication risk profiles in older adults receiving psychogeriatric outpatient care, with a specific focus on depressive episodes. In line with the exploratory nature of the study, we focused on two clinically motivated expectations: that medication load would be the main variable associated with potential DDI burden, and that depressive and anxiety overlap would reveal common DDI patterns involving sedative-hypnotics and anxiolytics/antidepressants. We also examined whether DDI and medication-risk burden features separated patients along a lower versus higher-burden prescribing risk gradient. The objectives were to: (i) describe the demographic, diagnostic, and medication-related profile of a psychogeriatric cohort in which depressive episodes were the dominant diagnosis; (ii) quantify DDI burden at visit and patient levels; (iii) identify recurrent interaction patterns among patients with depressive episodes and depressive/anxiety diagnostic overlap; (iv) examine multivariable factors associated with major and overall potential interaction burden; and (v) derive exploratory patient-level prescribing risk profiles relevant to medication review in late-life depression and assess their internal stability.

## 2 Material and methods

### 2.1 Study design and data source

We conducted a retrospective observational analysis of a dataset containing all eligible psychiatric visits from January 1 to December 31, 2023. Data were obtained from a single psychogeriatric outpatient clinic in Timişoara, Romania, where care was provided by multiple psychiatrists. Eligible visits were defined as those involving patients aged 65 years or older, with at least one psychiatric diagnosis, signed informed consent for participation and use of de-identified clinical data, and at least two prescribed drugs for visit-level pairwise DDI assessment. Visits not meeting the eligibility criteria above were excluded from the study. The structured medication list recorded at each visit included psychiatric and non-psychiatric treatment documented in the clinical record; therefore, the medication regimens analyzed were not restricted to psychotropic drugs.

The dataset was structured at the visit level, with one row per visit. Psychiatric diagnoses were recorded as 3-digit diagnosis codes based on the Romanian 999-code disease list, a national morbidity coding system aligned with the World Health Organization International Classification of Diseases, 10th Revision (ICD-10) 44. The subset of psychiatric 3-digit codes used in this study, together with English labels, is provided in **Supplementary Table 1**.

### 2.2 Study population and depression-focused analytic samples

The analytic cohort included adults aged 65 years or older with at least two prescribed drugs recorded at an eligible psychiatric outpatient visit. Patients were identified by a unique patient code, allowing repeated visits to be linked and aggregated for patient-level analyses. For the depression-focused analyses, the primary subgroup comprised patients with at least one depressive episode diagnosis (code 321) recorded during 2023. Analyses were performed at both the visit and patient levels. Because the study was designed to reflect real-world psychogeriatric outpatient prescribing, the main analytic cohort was not restricted exclusively to patients with depressive episodes. Instead, the full cohort was used to characterize the broader prescribing context for managing late-life depressive episodes, including psychiatric multimorbidity, cognitive disorders, anxiety disorders, and somatic co-treatment, and to derive overall prescribing risk profiles.

Because this was a retrospective census of eligible outpatient visits recorded during the study period, we did not perform a formal *a priori* sample size calculation. We included in the analysis all eligible records that met the inclusion criteria. No imputation was performed. All records included in the analytic cohort had complete age, sex, psychiatric diagnosis, and structured medication fields required for the main visit-level and patient-level analyses; therefore, no eligible visits were excluded because of missing data in these core variables.

Depressive episodes were identified using psychiatric diagnosis code 321 from the Romanian 999-code disease list (**Supplementary Table 1**). The primary depression subgroup was defined at the patient level and comprised patients with at least one depressive episode diagnosis code recorded during 2023. For visit-level descriptions, we separately reported all eligible visits of these patients and the subset of visits in which code 321 was recorded. Because anxiety symptoms and insomnia commonly complicate the pharmacological management of late-life depression, we also examined patients with anxiety disorder diagnosis code 325 and a same-visit depressive and anxiety overlap subgroup, defined as patients with at least one visit in which codes 321 and 325 were recorded together. Other affective disorder codes were not integrated into the primary depression subgroup unless code 321 was also recorded. Within these diagnosis-defined subgroups, major and moderate potential DDIs were ranked according to patient-level prevalence to identify recurrent interaction patterns relevant to late-life depression pharmacotherapy.

Patient-level regression analyses were performed in the depressive episode subgroup to identify determinants of DDI burden among older adults with depressive episodes. Profile analyses were performed in the full psychogeriatric cohort to preserve statistical stability and characterize the broader prescribing risk structure of the clinical setting in which late-life depressive episodes were treated. These analyses were interpreted alongside the depression-focused DDI rankings to identify medication review priorities relevant to depressive episodes in older adults.

All data were de-identified before analysis. The study received approval from the Scientific Research Ethics Committee of the “Victor Babeş” University of Medicine and Pharmacy Timişoara, Romania (approval no. 59/16.12.2022 rev). All participants provided written informed consent to participate in this study.

**Figure 1:**
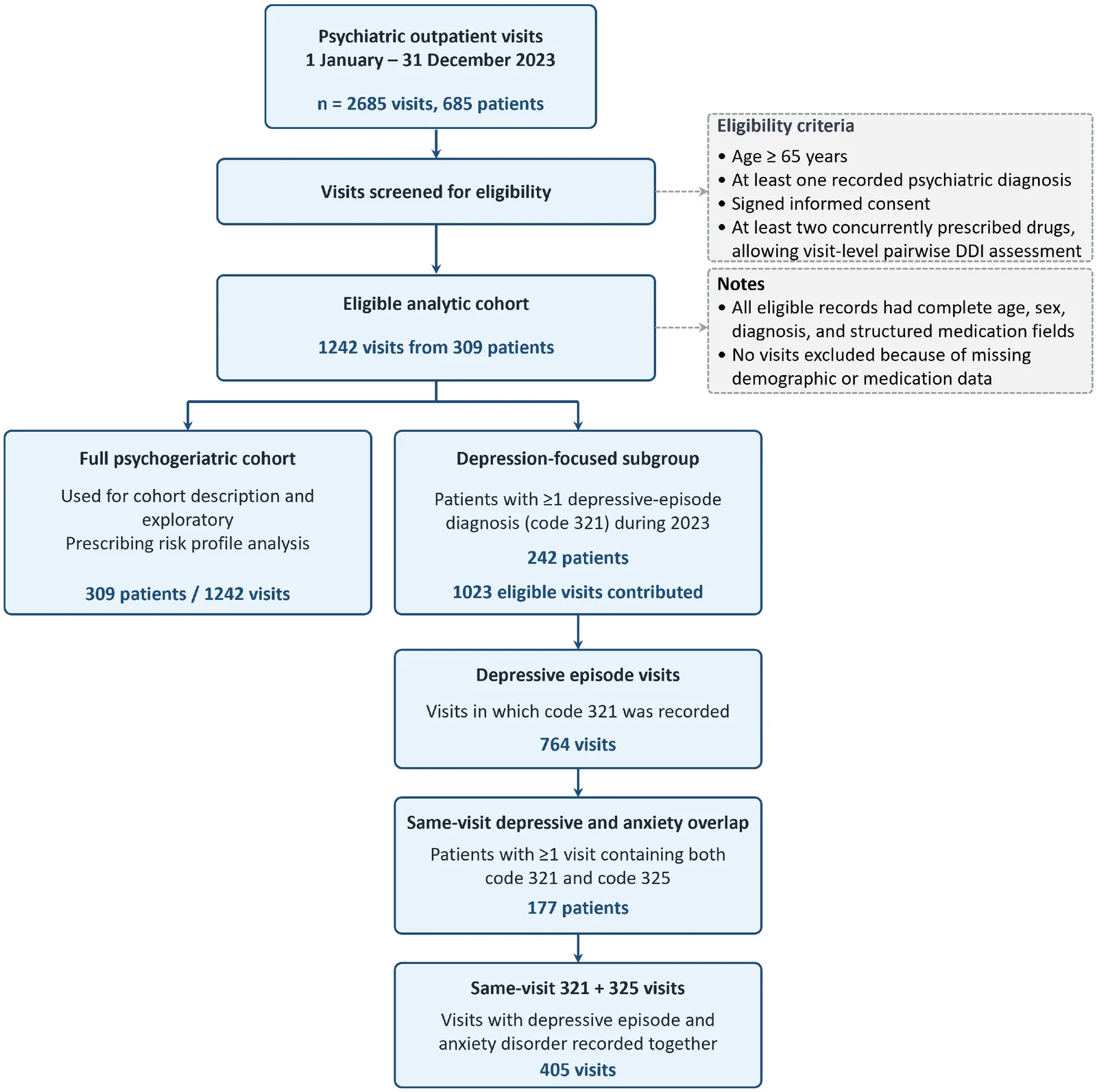
Analytic cohort construction and depression-focused denominators. Eligible visits were psychiatric outpatient visits recorded in 2023 involving patients aged 65 years or older, at least one psychiatric diagnosis, signed informed consent, and at least two prescribed drugs.

The construction of the analytic cohort and depression-focused analytic samples is summarized in **Figure 1**.

### 2.3 Drug exposure, DDI assessment, and medication risk burden measures

Prescribed drugs were extracted from the structured medication list recorded at each visit. Drug burden was quantified as the number of concurrent prescribed drugs per visit. For patient-level analyses, visit-level medication variables were summarized across all eligible visits during 2023 using mean values, maximum values, cumulative counts, or ever-exposure indicators, depending on the analytic objective.

We standardized drug names for DDI assessment by mapping Romanian entries to DrugBank substances. For fixed-dose combinations, we mapped the active substances separately. The final working medication file contained 228 unique standardized active substance entries after splitting fixed-dose combinations and final drug name standardization checks. All 228 entries (100.0%) were successfully mapped from Romanian medication names to the corresponding English DrugBank substance names; therefore, 0 entries (0.0%) remained unmapped after the standardization process. DDI counts were subsequently calculated using the standardized DrugBank-mapped active substance lists. We assessed potential DDIs using DrugBank Online/API 5.1.11 Knox et al. (45). For each visit, all possible pairwise combinations of concurrently prescribed drugs were generated and evaluated. DrugBank classifies DDIs as major, moderate, minor, or no interaction. All identified DDIs represent potential and not confirmed adverse events. Visit-level DDI burden was quantified using severity-specific counts, including the number of major, moderate, and minor DDI pairs. We defined total DDI burden as the sum of major, moderate, and minor DDI pairs, thereby excluding drug pairs classified as having no interaction. For descriptive analyses, we also derived binary visit-level indicators for the presence of at least one major DDI, at least one moderate DDI, and at least one moderate-or-major DDI.

In addition to DDI counts, we quantified regimen-level medication risk burden using predefined drug-level crosswalks for anticholinergic burden, QT/TdP risk, potentially inappropriate prescribing in older adults, and syndrome of inappropriate antidiuretic hormone secretion (SIADH) or hyponatremia risk. Anticholinergic burden was coded using the Anticholinergic Cognitive Burden (ACB) framework, with drugs assigned a score of 1 for possible anticholinergic activity and 2 or 3 for definite anticholinergic activity; drugs not listed in the framework were assigned a score of 0 King and Rabino (46). QT/TdP risk drug burden was derived from the CredibleMeds QTdrugs List (accessed on October 31, 2025), maintained by the Arizona Center for Education and Research on Therapeutics Woosley et al. (28). For visit-level aggregation, drugs classified as having known risk of torsades de pointes were assigned a score of 3, drugs with possible risk were assigned a score of 2, and drugs with conditional risk were assigned a score of 1; drugs not included in these categories were assigned a score of 0. Potentially inappropriate medication burden was coded using the 2023 American Geriatrics Society Beers Criteria American Geriatrics Society (31). Drugs categorized as potentially inappropriate in older adults were assigned a score of 3, drugs considered potentially inappropriate in specific drug-disease or drug-syndrome contexts were assigned a score of 2, and drugs recommended for use with caution in older adults were assigned a score of 1. Drugs not mapped to these categories were assigned a score of 0. SIADH risk exposure was coded as a binary drug-level indicator, with drugs considered to carry SIADH or hyponatremia risk coded as 1 and all other drugs coded as 0 Knox et al. (45).

For each visit, drug-level scores were summed across the prescribed regimen to generate cumulative visit-level burden measures. The ACB, QT/TdP risk, and Beers burden variables, therefore, represent the sum of ordinal drug-level risk scores within a visit. SIADH risk drug burden represents the count of concurrently prescribed SIADH risk drugs within a visit.

DDI burden was analyzed at both the visit and patient levels. Visit-level outcomes included the number of total DDI pairs, the presence of any DDI, the presence of any moderate-or-major DDI, and the presence of any major DDI. Patient-level outcomes included the cumulative annual number of DDI pairs, mean DDI burden per visit, maximum DDI burden in any visit, and ever exposure to at least one major DDI during 2023.

For depression-focused analyses, the main descriptive outcomes were DDI burden and DDI-pair rankings among patients with depressive episodes and among patients with depressive and anxiety overlap. For multivariable patient-level models, the binary outcome was the occurrence of at least one major DDI during 2023. Count-based outcomes included annual total DDI pairs and annual major DDI pairs.

### 2.4 Multivariable modeling of DDI burden

Patient-level multivariable models were used to examine factors associated with potential DDI burden. For the binary outcome of any major DDI during 2023, we fitted logistic regression models. For count-based DDI outcomes, we used negative binomial regression models because DDI counts were overdispersed. For negative binomial models, the cumulative annual number of potential DDI pairs was modeled as the outcome, and the natural logarithm of the number of eligible visits during 2023 was included as an offset with coefficient fixed at 1. This offset accounted for differences in the number of outpatient observation and prescribing opportunities per patient and allowed the models to estimate visit-adjusted annual potential DDI-pair rates instead of unadjusted cumulative counts.

The main regression analyses were performed in the depressive-episode subgroup to identify determinants of DDI burden among older patients with depressive episodes. Candidate predictors included sex, age at first visit, number of visits during 2023, mean number of drugs per visit, number of unique psychiatric diagnoses, anxiety disorder diagnosis, vascular dementia, other mental disorders due to brain injury, brain dysfunction, or somatic condition, acute and transient psychotic disorders, and bipolar affective disorder. Model specification was adapted to the number of events and distribution of predictors in the depressive-episode subgroup to avoid overfitting.

Results were reported as odds ratios or incidence rate ratios with 95% confidence intervals. Logistic model discrimination was summarized using the area under the receiver operating characteristic curve (AUC).

Model complexity was limited according to the number of patients and outcome events. Diagnosis-extended models were restricted to common co-diagnoses and were interpreted as exploratory.

Approximate linearity of continuous predictors in the model scale was assessed by refitting the regression models with centered quadratic terms for the continuous predictors included in each model. The diagnostic quadratic models were compared with the retained linear-term models using Akaike information criterion (AIC) and likelihood-ratio testing. Multicollinearity was evaluated using variance inflation factors (VIFs). Influential observations were assessed using standardized Pearson residuals, leverage, and Cook’s distance, followed by exclusion sensitivity analyses in which models were refitted after removing observations flagged by these criteria. For count outcomes, overdispersion was assessed by comparing Poisson and negative binomial models with the same predictors and visit-number offset, using Pearson dispersion and AIC. These analyses were used as model diagnostics and sensitivity checks, while the primary models retained linear terms to preserve interpretability of odds ratios and incidence rate ratios.

### 2.5 Exploratory prescribing risk profile analysis

To identify patient-level prescribing risk profiles, we built a patient-level matrix using DDI and medication risk burden features as active variables. These included the mean number of drugs per visit; mean total, major, moderate, and minor DDI pairs per visit; proportions of visits with any major DDI and any total DDI; mean ACB, QT/TdP risk, Beers, and SIADH risk drug burden per visit; maximum number of drugs in any visit; and maximum number of major DDI pairs in any visit.

We performed the primary profile analysis in the full psychogeriatric cohort to preserve sample size and capture the real-world prescribing risk structure of the clinical setting in which late-life depressive episodes were treated. The multivariate structure was visualized using principal component analysis (PCA). K-means clustering was then applied to the standardized patient-level burden matrix, and candidate two-, three-, and four-cluster solutions were examined. The retained solution was selected based on separation, stability, cluster-size balance, and clinical interpretability.

The retained profile solution was evaluated for internal stability using repeated random starts, 80% patient subsampling, and leave-one-feature-out sensitivity analyses. Concordance with the baseline partition was quantified using the adjusted Rand index and mapped agreement. To assess clinical relevance and avoid circularity, the profiles were externally characterized using variables not included as active clustering features, including sex, age at first visit, number of visits during 2023, number of unique psychiatric diagnoses, diagnosis-burden categories, and indicators for common psychiatric diagnoses.

For the depression-focused adaptation, profile membership was described among patients with depressive episodes and among those with depressive and anxiety overlap. This analysis evaluated whether older patients with depressive episodes were preferentially represented in the higher burden prescribing risk profile and whether the profile structure captured clinically relevant medication review priorities for late-life depression.

### 2.6 Statistical analysis

All tests were two-sided and *p <*0.05 was considered statistically significant. Because sex-stratified, diagnosis-specific subgroup, and prescribing risk profile comparisons were exploratory, *p*-values were interpreted descriptively. Analyses were conducted in Python using standard data management, regression, and machine-learning workflows.

## 3 Results

### 3.1 Cohort profile, depressive-episode analytic subgroup, and psychiatric co-diagnosis patterns

The final analytic cohort comprised 1242 psychiatric outpatient visits from 309 adults aged 65 years or older with at least two prescribed drugs during 2023. Depressive episodes were the dominant diagnostic category, identified in 242 patients, corresponding to 78.3% of the full cohort. These patients contributed 1023 visits during the study year, of which 764 visits included a depressive episode code in the visit record. Anxiety disorders were also common, affecting 232 patients, and 177 patients had at least one same-visit co-occurrence of depressive episode and anxiety disorder codes.

The depressive-episode subgroup was broadly similar to the full cohort in age and sex distribution, but showed slightly greater psychiatric diagnostic complexity. Women represented 182 of 242 patients with depressive episodes (75.2%), and the median age at the first visit was 75 years [69–82]. Patients with depressive episodes had a mean of 2.81 unique psychiatric diagnoses during 2023, compared to 2.61 in the full cohort. In patients with depressive episodes, 218 (90.1%) had at least two psychiatric diagnoses, while 120 (49.6%) had at least three psychiatric diagnoses. **Table 1** summarizes patient-level demographic, diagnostic, medication-exposure, DDI-burden, and medication risk characteristics for the full cohort and depressive-episode subgroup.

**Table 1:** Patient-level characteristics of the full cohort and depressive-episode subgroup.

Characteristic	Full cohort	Depressive-episode subgroup
Patients, *n*	309	242
Eligible visits, *n*	1242	1023
Women, *n* (%)	227 (73.5%)	182 (75.2%)
Men, *n* (%)	82 (26.5%)	60 (24.8%)
Age at first visit, years	76.11 (7.74); 75 [70–82]	75.95 (7.87); 75 [69–82]
Visits/patient	4.02 (3.49); 3 [1–6]	4.23 (3.52); 3 [1–6]
Mean drugs/visit	5.97 (2.83)	6.09 (2.81)
Unique psychiatric diagnoses	2.61 (1.30); 2 [2–3]	2.81 (1.29); 2 [2–4]
Patients with ≥2 unique psychiatric diagnoses, *n* (%)	254 (82.2%)	218 (90.1%)
Patients with ≥3 unique psychiatric diagnoses, *n* (%)	134 (43.4%)	120 (49.6%)
Patients with ≥1 major DDI, *n* (%)	158 (51.1%)	134 (55.4%)
Patients with ≥1 moderate DDI, *n* (%)	287 (92.9%)	230 (95.0%)
Patients with ≥1 moderate-or-major DDI, *n* (%)	290 (93.9%)	233 (96.3%)
Total identified potential DDI pairs	47.13 (64.21); 22 [6–63]	50.57 (65.28); 25 [8–67]
Mean ACB burden/visit	1.94 (1.65)	1.95 (1.64)
Mean QT/TdP risk drug burden/visit	1.98 (1.39)	2.07 (1.39)
Mean Beers burden/visit	5.43 (2.97)	5.63 (2.97)
Mean SIADH risk drugs/visit	0.90 (0.79)	0.95 (0.80)

ACB, Anticholinergic Cognitive Burden; DDI, drug-drug interaction; IQR, interquartile range; QT/TdP, QT prolongation/torsades de pointes; SD, standard deviation; SIADH, syndrome of inappropriate antidiuretic hormone secretion.

Values are presented as mean (SD), median [IQR], or *n* (%). The depressive-episode subgroup includes patients with at least one depressive episode diagnosis code recorded during 2023. Visit-level variables in this table were first summarized within patients and then summarized across patients. Total identified potential DDI pairs represent the sum of major, moderate, and minor DrugBank-identified potential DDI pairs, excluding drug pairs classified as having no interaction.

**Table 2** shows the patterns of psychiatric co-diagnosis among patients with depressive episodes. Anxiety disorders were the dominant co-occurring diagnosis, affecting 193 of 242 patients with depressive episodes (79.8%) and occurring in the same visit as a depressive episode in 177 patients (73.1%). At the visit level, anxiety disorders were recorded in 405 of 764 depressive-episode visits (53.0%). Other frequent co-occurring diagnoses included other organic/somatic mental disorders, vascular dementia, acute and transient psychotic disorders, affective mood disorders, bipolar affective disorder, and non-organic sleep disorders. These findings indicate that late-life depressive episodes were managed in a complex psychogeriatric context, characterized by prominent anxiety, cognitive, psychotic, mood, and sleep-related comorbidities.

**Table 2:** Top ten co-occurring psychiatric diagnoses among patients with depressive episodes.

Co-occurring psychiatric diagnosis	Patients, annual, *n* (%)	Patients, same-visit, *n* (%)	Depressive episode visits, *n* (%)
Anxiety disorders	193 (79.8%)	177 (73.1%)	405 (53.0%)
Other organic/somatic mental disorders	59 (24.4%)	42 (17.4%)	111 (14.5%)
Vascular dementia	51 (21.1%)	31 (12.8%)	71 (9.3%)
Acute and transient psychotic disorders	38 (15.7%)	25 (10.3%)	52 (6.8%)
Affective mood disorders	25 (10.3%)	20 (8.3%)	39 (5.1%)
Bipolar affective disorder	21 (8.7%)	7 (2.9%)	11 (1.4%)
Non-organic sleep disorders	20 (8.3%)	11 (4.5%)	30 (3.9%)
Schizophrenia	11 (4.5%)	7 (2.9%)	14 (1.8%)
Persistent delusional disorder	5 (2.1%)	3 (1.2%)	3 (0.4%)
Schizoaffective disorders	5 (2.1%)	1 (0.4%)	11 (1.4%)

Percentages in the first two columns use depressive-episode patients as the denominator (n=242). The first column lists diagnoses from any eligible visit during 2023. The second column refers to patients who had at least one visit where both code 321 and the co-occurring diagnosis were recorded. Percentages in the third column use depressive-episode visits as the denominator (n=764), defined as visits in which code 321 was recorded. Multiple diagnosis categories could be recorded for the same patient or visit; therefore, percentages do not sum to 100%. Other organic/somatic mental disorders refers to other mental disorders due to brain injury, brain dysfunction, or somatic condition.

The annual patient-level column includes diagnoses recorded at any eligible visit during 2023 among patients with depressive episodes. The same-visit patient-level column includes patients with at least one visit in which the co-occurring diagnosis and depressive episode were recorded together.

Exploratory sex-stratified analyses among patients with depressive episodes showed that men had higher medication load than women (mean drugs/visit 6.76 vs. 5.87, *p* = 0.029) and higher total DDI burden (mean total potential DDI pairs/visit 13.67 vs. 10.10, *p* = 0.013; annual total potential DDI pairs 66.92 vs. 45.19, *p* = 0.031). The psychiatric diagnostic burden was similar (unique psychiatric diagnoses 2.90 vs. 2.79, *p* = 0.653). Major DDI exposure and higher-burden profile membership were numerically higher in men but did not reach conventional statistical significance (patients with ≥1 major DDI 66.7% vs. 51.6%, *p* = 0.060; higher-burden profile 45.0% vs. 32.4%, *p* = 0.107). The full sex-stratified comparison of demographic, diagnostic, DDI burden, medication risk, and profile variables among patients with depressive episodes is provided in **Supplementary Table 2**.

### 3.2 DDI burden in the depressive-episode subgroup

The DDI burden at the visit level was high among patients with depressive episodes, particularly for moderate interactions (**Table 3**). Across all visits contributed by depressive episode patients, major DDIs occurred in approximately half of the visits, while moderate DDIs and moderate-or-major DDIs occurred in most visits. Compared with the full cohort, visits contributed by depressive episode patients showed slightly higher drug exposure, major-DDI prevalence, and overall DDI burden.

**Table 3:** Visit-level DDI and medication risk burden in the full cohort, depressive-episode subgroup, and same-visit depressive and anxiety overlap visits.

Characteristic	Full cohort	Depressive-episode subgroup	Same-visit 321 + 325 visits
Visits, *n*	1242	1023	405
Drugs/visit	6.35 (3.00); 6 [4–8]	6.44 (2.97); 6 [4–8]	6.55 (2.99); 6 [4–9]
Visits with ≥1 major DDI, *n* (%)	602 (48.5%)	516 (50.4%)	181 (44.7%)
Visits with ≥1 moderate DDI, *n* (%)	1141 (91.9%)	959 (93.7%)	399 (98.5%)
Visits with ≥1 moderate-ormajor DDI, *n* (%)	1161 (93.5%)	971 (94.9%)	400 (98.8%)
Total potential DDI pairs/visit	11.72 (10.77); 9 [3–17]	11.96 (10.41); 9 [4–17]	12.88 (10.92); 10 [4–18]
Major DDI pairs/visit	1.15 (1.68)	1.23 (1.73)	1.12 (1.67)
Moderate DDI pairs/visit	6.11 (5.78)	6.18 (5.50)	7.03 (5.76)
Minor DDI pairs/visit	4.47 (5.05)	4.56 (4.93)	4.73 (5.14)
ACB burden/visit	2.15 (1.95)	2.12 (1.93)	2.18 (1.66)
QT/TdP risk burden/visit	2.05 (1.54)	2.12 (1.57)	1.92 (1.46)
Beers burden/visit	5.74 (3.20)	5.84 (3.18)	6.49 (3.21)
SIADH risk drugs/visit	0.99 (0.84)	1.00 (0.83)	0.90 (0.77)

ACB, Anticholinergic Cognitive Burden; DDI, drug-drug interaction; IQR, interquartile range; QT/TdP, QT prolongation/torsades de pointes; SD, standard deviation; SIADH, syndrome of inappropriate antidiuretic hormone secretion.

Values are presented as mean (SD), median [IQR], or *n* (%). The depressive-episode patients column includes all eligible visits from patients with at least one depressive episode diagnosis code 321 during 2023. Same-visit 321 + 325 visits are visits in which depressive episode code 321 and anxiety disorder code 325 were recorded together. Percentages use the number of visits in each column as the denominator.

**Figure 2:**
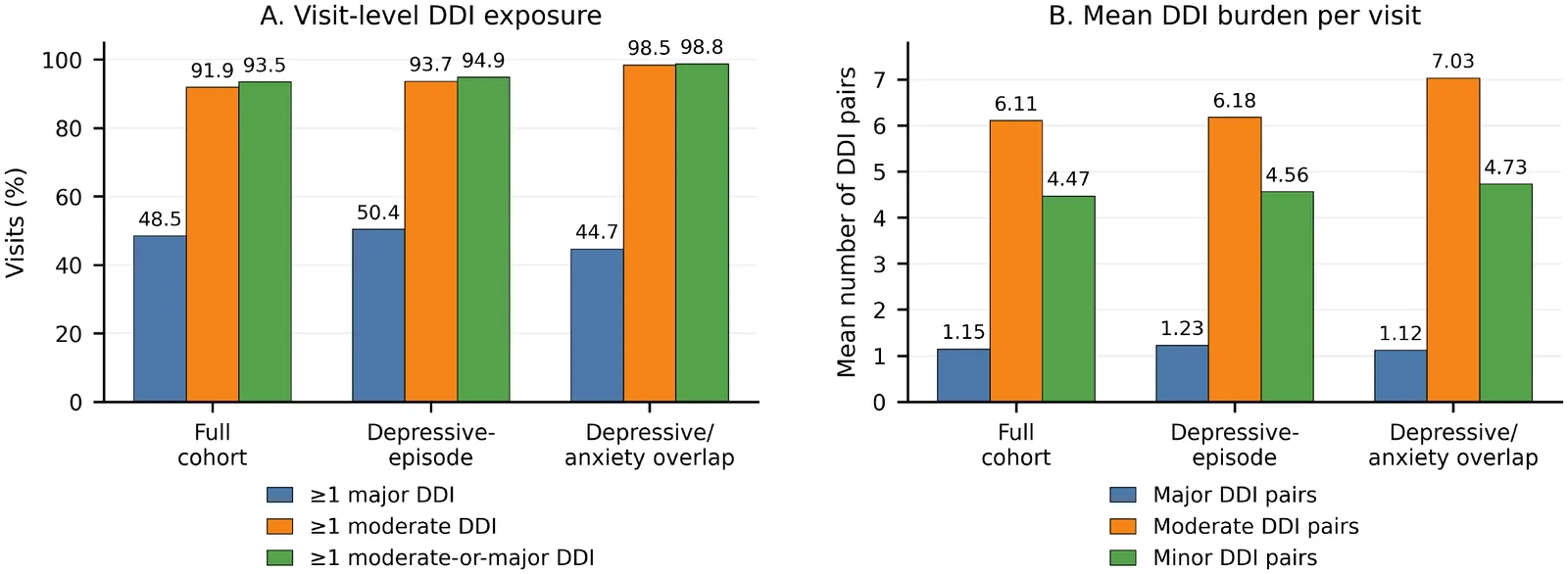
Visit-level drug-drug interaction (DDI) burden across analytic subgroups. **(A)** Percentage of visits with at least one major DDI, at least one moderate DDI, and at least one moderate-or-major DDI in the full cohort, depressive-episode subgroup, and same-visit depressive/anxiety overlap visits. **(B)** Mean number of major, moderate, and minor DDI pairs per visit across the same analytic subgroups. The depressive/anxiety overlap subgroup refers to visits in which depressive episode and anxiety disorder diagnoses were recorded together.

**Figure 2** summarizes visit-level DDI burden across analytic subgroups, showing consistently high moderate and moderate-or-major DDI exposure and higher mean moderate-DDI burden in same-visit depressive and anxiety overlap visits.

The subgroup of patients with same-visit depressive and anxiety overlap showed the highest level of moderate DDIs. In visits where depressive episode and anxiety disorder codes were recorded together, moderate DDIs and moderate-or-major DDIs were almost universal, while the average number of moderate DDI pairs per visit was higher than in both the full cohort and the broader depressive-episode subgroup.

Medication risk burden was also evident across depression-related visits, including anticholinergic, QT/TdP risk, Beers, and SIADH-related exposure.

### 3.3 Diagnosis-specific DDI patterns relevant to late-life depression

Patient-level DDI rankings showed that interaction burden among older adults with depressive episodes was not limited to antidepressant-centered combinations (**Table 4**). Across the depressive episode, anxiety disorder, and same-visit depressive and anxiety overlap subgroups, the leading major DDI pairs included cardiovascular combinations and pairs involving carbamazepine or trazodone. In contrast, the leading moderate DDI pairs were mainly benzodiazepine/Z-drug and benzodiazepine/antidepressant combinations.

**Table 4:** Leading patient-level major and moderate potential DDI pairs in depressive-episode, anxietydisorder, and same-visit depressive and anxiety overlap subgroups.

Subgroup	Severity	Leading DDI pairs, patients *n* (%)
Depressive episode (*n* = 242 patients)	Major	atorvastatin + clopidogrel, 16 (6.6%) indapamide + trazodone, 13 (5.4%) alprazolam + carbamazepine, 12 (5.0%)
	Moderate	alprazolam + zolpidem, 23 (9.5%) lorazepam + zolpidem, 23 (9.5%) lorazepam + mirtazapine, 19 (7.9%) lorazepam + trazodone, 19 (7.9%)
Anxiety disorders (*n* = 232 patients)	Major	atorvastatin + clopidogrel, 15 (6.5%) alprazolam + carbamazepine, 13 (5.6%) atorvastatin + carbamazepine, 12 (5.2%)
	Moderate	lorazepam + zolpidem, 28 (12.1%) alprazolam + zolpidem, 26 (11.2%) alprazolam + trazodone, 19 (8.2%) lorazepam + mirtazapine, 19 (8.2%) lorazepam + trazodone, 19 (8.2%)
Same-visit 321 + 325 overlap (*n* = 177 patients)	Major	atorvastatin + clopidogrel, 10 (5.6%) indapamide + trazodone, 8 (4.5%) atorvastatin + carbamazepine, 7 (4.0%) bisoprolol + spironolactone, 7 (4.0%)
	Moderate	lorazepam + trazodone, 19 (10.7%) alprazolam + zolpidem, 18 (10.2%) lorazepam + mirtazapine, 18 (10.2%) lorazepam + zolpidem, 16 (9.0%)

Percentages use the number of patients in each subgroup as the denominator. The depressive-episode subgroup includes patients with at least one depressive episode diagnosis code 321 recorded during 2023. The anxiety-disorder subgroup includes patients with at least one anxiety disorder diagnosis code 325 recorded during 2023. The same-visit 321 + 325 overlap subgroup includes patients with at least one visit in which depressive episode code 321 and anxiety disorder code 325 were recorded together.

This pattern was especially clinically relevant in the same-visit depressive and anxiety overlap subgroup, where recurrent moderate DDI pairs included lorazepam + trazodone, alprazolam + zolpidem, lorazepam + mirtazapine, and lorazepam + zolpidem. The full diagnosis-specific patient-level rankings are provided in **Supplementary Table 3**.

### 3.4 Multivariable factors associated with potential DDI burden among patients with depressive episodes

Among patients with depressive episodes, medication load was the most consistent factor associated with potential DDI burden across all multivariable models (**Table 5**). In the logistic regression model for any major potential DDI during 2023, each additional mean drug per visit was associated with 57% higher odds of major DDI exposure (OR 1.57, 95% CI 1.37–1.81, *p <* 0.001). Each additional visit during 2023 was also independently associated with major potential DDI exposure (OR 1.18, 95% CI 1.05–1.33, *p* = 0.007), consistent with greater opportunity for cumulative exposure across repeated outpatient contacts. In contrast, the number of unique psychiatric diagnoses was not independently associated with any major potential DDI in the binary model. The logistic regression model for any major potential DDI during 2023 showed good discrimination, with an AUC of 0.833 for the core model and 0.834 for the diagnosis-extended model.

**Table 5:** Multivariable factors associated with DDI burden among patients with depressive episodes.

Model specification	Predictor	Estimate (95% CI)	*p*-value
Panel A. Logistic regression model for any major potential DDI during 2023			
Core model	Mean drugs per visit	1.57 (1.37–1.81)	*<*0.001
Core model	Visits during 2023	1.18 (1.05–1.33)	0.007
Core model	Unique psychiatric diagnoses	1.18 (0.85–1.64)	0.319
Diagnosis-extended model	Anxiety disorders	0.85 (0.38–1.91)	0.690
Diagnosis-extended model	Vascular dementia	0.84 (0.36–1.92)	0.673
Diagnosis-extended model	Other organic/somatic mental disorders	1.10 (0.48–2.55)	0.821
Diagnosis-extended model	Acute and transient psychotic disorders	1.68 (0.61–4.64)	0.314
Diagnosis-extended model	Bipolar affective disorder	1.13 (0.36–3.50)	0.835
Panel B. Negative binomial model for annual total potential DDI-pair rate			
Core model	Mean drugs per visit	1.35 (1.32–1.38)	*<*0.001
Core model	Unique psychiatric diagnoses	1.03 (0.99–1.06)	0.142
Diagnosis-extended model	Anxiety disorders	1.16 (1.02–1.31)	0.023
Diagnosis-extended model	Vascular dementia	0.90 (0.80–1.00)	0.060
Diagnosis-extended model	Other organic/somatic mental disorders	0.98 (0.88–1.10)	0.789
Diagnosis-extended model	Acute and transient psychotic disorders	1.06 (0.94–1.21)	0.342
Diagnosis-extended model	Bipolar affective disorder	1.07 (0.92–1.25)	0.383
Panel C. Negative binomial model for annual major potential DDI-pair rate			
Core model	Mean drugs per visit	1.38 (1.28–1.48)	*<*0.001
Core model	Unique psychiatric diagnoses	1.19 (1.05–1.35)	0.008
Diagnosis-extended model	Anxiety disorders	0.92 (0.58–1.45)	0.717
Diagnosis-extended model	Vascular dementia	0.95 (0.62–1.46)	0.830
Diagnosis-extended model	Other organic/somatic mental disorders	1.59 (1.05–2.41)	0.027
Diagnosis-extended model	Acute and transient psychotic disorders	1.11 (0.71–1.76)	0.642
Diagnosis-extended model	Bipolar affective disorder	1.61 (0.92–2.81)	0.094

Estimates are from patient-level models fitted in the depressive-episode subgroup (*n* = 242). Values in Panel A are odds ratios (ORs) from the logistic regression model for any major potential DDI during 2023. Values in Panels B and C are incidence rate ratios (IRRs) from negative binomial models for annual total potential DDI-pair rate and annual major potential DDI-pair rate, respectively. Negative binomial models used the natural logarithm of the number of eligible visits during 2023 as an offset, so estimates refer to annual potential DDI-pair rates rather than unadjusted cumulative counts. The diagnosis-extended rows represent models adding the listed diagnosis indicators to the core covariates. *p*-values are interpreted descriptively in these exploratory models.

In the negative binomial rate models, mean drug exposure remained strongly associated with both annual total potential DDI burden and annual major potential DDI burden. Anxiety disorders were associated with a higher annual total potential DDI rate, and the number of unique psychiatric diagnoses was independently associated with the annual major potential DDI rate. Other mental disorders due to brain injury, brain dysfunction, or somatic condition were also associated with higher annual major potential DDI rates in the diagnosis-extended model, while bipolar affective disorder showed a non-significant trend in the same direction.

Model diagnostics supported the retained regression approach while also reinforcing the exploratory interpretation of the patient-level models. Quadratic term sensitivity analyses did not improve the logistic models for any major potential DDI during 2023 (core model: ΔAIC=5.11, likelihood ratio *p* = 0.576; diagnosis-extended model: ΔAIC=3.14, likelihood ratio *p* = 0.413), whereas negative binomial rate models showed evidence of non-linearity mainly related to medication load. Multicollinearity was low across models, with maximum VIF values ranging from 1.065 to 1.745. Influence diagnostics identified flagged observations, but the main medication load association remained stable, with mean drugs per visit positively associated with potential DDI burden in all models after exclusion sensitivity analyses. Overdispersion diagnostics supported negative binomial regression for count outcomes: Poisson models showed Pearson dispersion values of 4.42–4.78, whereas negative binomial models reduced dispersion to 0.75–0.91 and improved AIC by 566.7–658.1 points.

### 3.5 Exploratory prescribing risk profiles and relevance to depressive episodes

We performed exploratory risk profiling in the full psychogeriatric cohort using patient-level DDI- and medication risk burden features as active clustering variables. Among the candidate two-, three-, and four-cluster solutions, the two-cluster solution was retained because it provided the clearest lower- versus higher-burden gradient while preserving cluster-size balance and interpretability. The two-cluster solution had a silhouette coefficient of 0.316, indicating modest separation of the cohort into a lower-burden profile comprising 207 patients (67.0%) and a higher-burden profile comprising 102 patients (33.0%) (**Table 6**).

**Table 6:** Prescribing risk profiles in the full cohort and depression-focused subgroups.

Characteristic	Lower-burden blueprofile	Higher-burden blueprofile
Patients, *n* (%)	207 (67.0%)	102 (33.0%)
Depressive-episode patients, *n* (%)	156 (64.5%)	86 (35.5%)
Same-visit 321 + 325 overlap patients, *n* (%)	117 (66.1%)	60 (33.9%)
Mean drugs/visit	4.52	8.92
Mean bluetotal potential DDI pairs/visit	5.32	21.00
Mean major DDI pairs/visit	0.34	2.14
Mean moderate DDI pairs/visit	2.93	10.29
Mean minor DDI pairs/visit	2.05	8.57
Proportion of visits with major DDI	0.23	0.77
Proportion of visits with any bluepotential DDI	0.93	1.00
Mean ACB burden/visit	1.54	2.75
Mean QT/TdP risk burden/visit	1.59	2.78
Mean Beers burden/visit	4.43	7.47
Mean SIADH risk drugs/visit	0.72	1.25
Maximum drugs in any visit	5.30	10.43
Maximum major DDI pairs in any visit	0.56	3.10

ACB, Anticholinergic Cognitive Burden; DDI, drug-drug interaction; QT/TdP, QT prolongation/torsades de pointes; SIADH, syndrome of inappropriate antidiuretic hormone secretion.

The higher-burden profile showed a broad increase across medication risk dimensions compared to the lower-burden profile, with twice the mean drug exposure and nearly four times the mean number of total potential DDI pairs per visit. It also exhibited higher major, moderate, and minor DDI burden, along with higher anticholinergic, QT/TdP risk, and Beers medication risk burdens. PCA supported this interpretation, with PC1 explaining 49.3% of the variance and PC2 14.1%, highlighting a dominant polypharmacy and interaction-burden axis.

Depressive-episode patients were represented in both profiles. Among 242 patients with depressive episodes, 156 (64.5%) belonged to the lower-burden profile and 86 (35.5%) to the higher-burden profile. Among 177 patients with same-visit depressive and anxiety overlap, 117 (66.1%) belonged to the lower-burden profile and 60 (33.9%) to the higher-burden profile. This profile structure did not identify a specific subgroup for depression, but it revealed a gradient of prescribing risks within the care setting for managing late-life depressive episodes.

**Figure 3:**
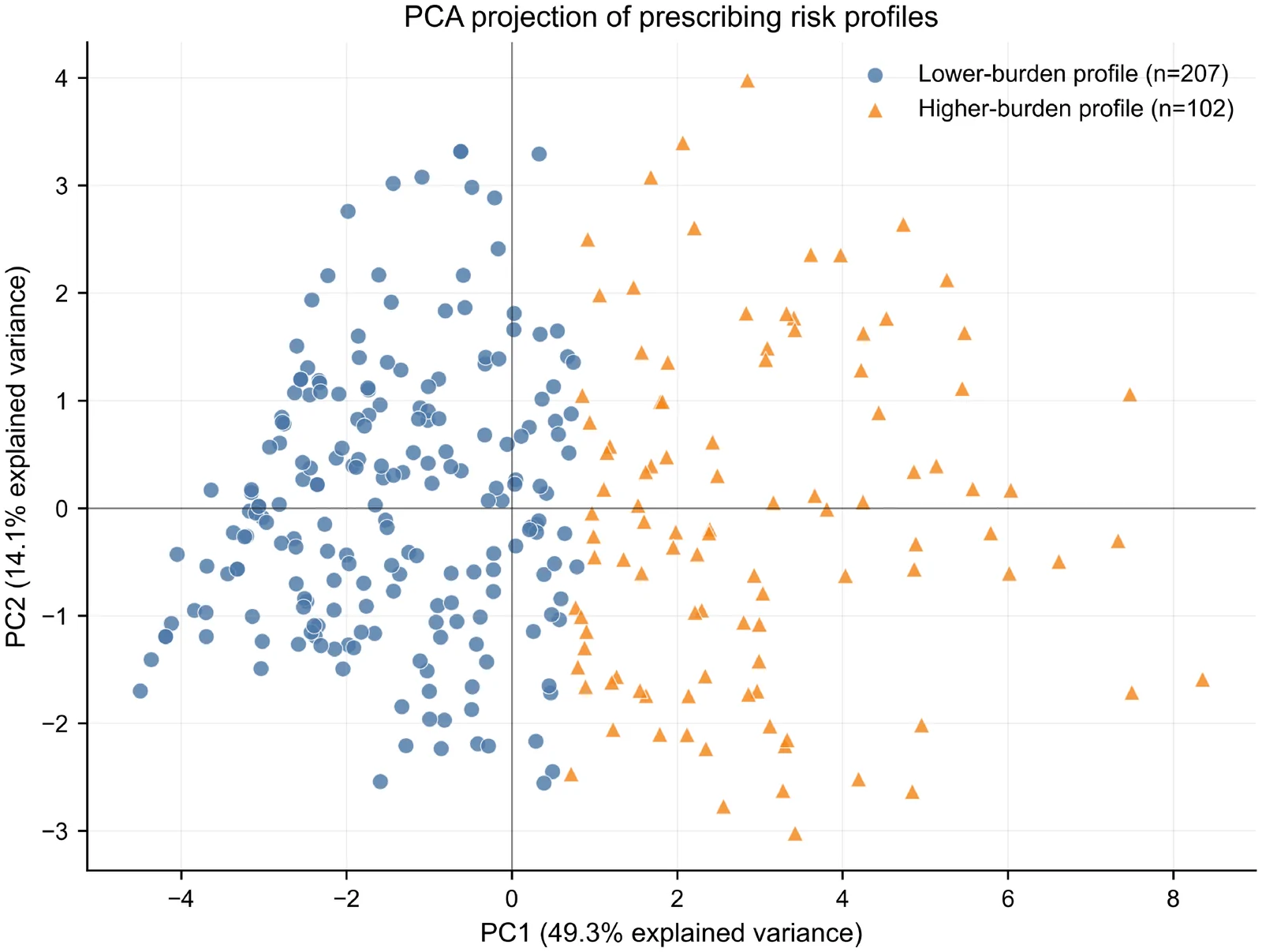
Principal component analysis of patient-level prescribing risk features by retained profile. Patients are projected onto the first two principal components derived from standardized DDI and medication risk burden features. Points represent individual patients, colored according to the retained prescribing risk profile. PC1 explained 49.3% of the variance and PC2 explained 14.1%, supporting a dominant prescribing risk axis separating lower- and higher-burden profiles.

The retained two-cluster solution showed acceptable robustness in sensitivity analyses. In 80% patient subsampling, the mean adjusted Rand index (ARI) was 0.857, and the median mapped agreement was 0.964. Leave-one-feature-out analyses showed similar stability, with a mean ARI of 0.837 and a median mapped agreement of 0.958. Repeated random-start analyses showed high median stability, although occasional single-initialization solutions diverged, consistent with the use of *n*_init_ =1 in this stress-test procedure. These analyses should be interpreted as internal stability checks and not as external clinical validation. The separation of the two retained profiles was also visible in the PCA projection, where PC1 captured the dominant prescribing risk gradient across medication load, DDI burden, and medication risk scores (**Figure 3**).

**Figure 4:**
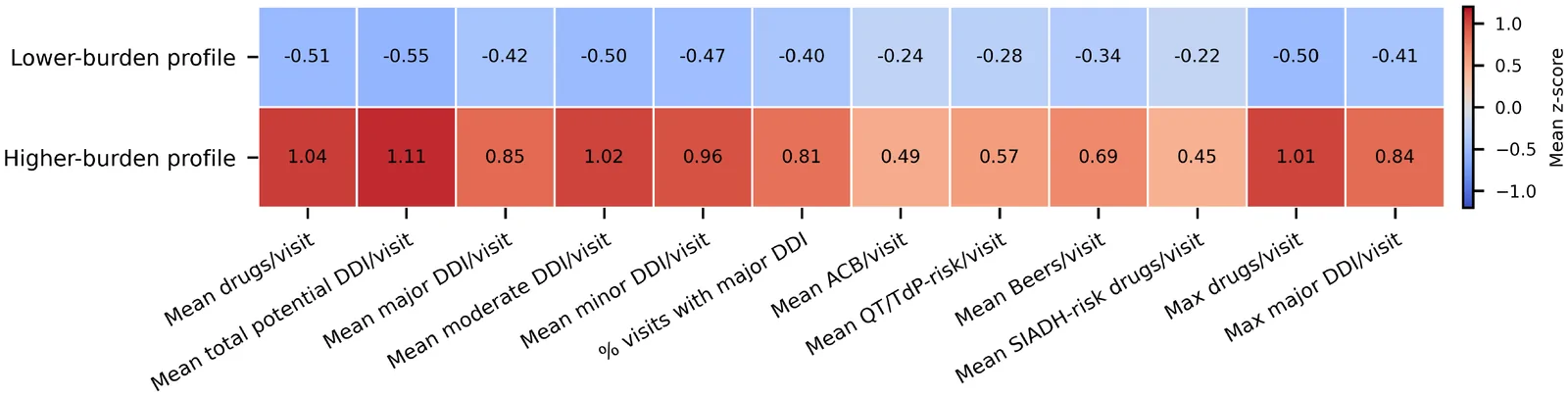
Heatmap of patient-level prescribing risk burden by retained profiles. The heatmap shows mean standardized values of active patient-level DDI and medication risk burden features in the lower-burden and higher-burden prescribing risk profiles. Values are expressed as *z*-scores relative to the full patient cohort. Negative values indicate below-average burden and positive values indicate above-average burden. (ACB, Anticholinergic Cognitive Burden; DDI, drug-drug interaction; QT/TdP, QT prolongation/torsades de pointes; SIADH, syndrome of inappropriate antidiuretic hormone secretion).

The profile heatmap in **Figure 4** further illustrates the multidimensional separation between the retained profiles: the higher-burden profile consistently showed higher values across polypharmacy, DDI burden, major DDI exposure, anticholinergic burden, QT/TdP risk burden, Beers burden, SIADH risk drug burden, and peak exposure measures.

## 4 Discussion

### 4.1 Overview of findings

In our retrospective psychogeriatric outpatient cohort, older adults with depressive episodes experienced substantial DDI burden in the context of high psychiatric co-diagnosis burden, polypharmacy, and cumulative geriatric medication risk exposure. Depressive episodes were the dominant diagnostic category, affecting 242 of 309 patients, and most patients with depressive episodes had at least two psychiatric diagnoses during the study year. Anxiety disorders were the most frequent co-occurring diagnosis, affecting nearly 80% of patients with depressive episodes and co-occurring with a depressive episode at the same visit in more than 70% of them. Other recorded psychiatric co-diagnoses included organic/somatic mental disorders, vascular dementia, psychotic-spectrum disorders, affective mood disorders, bipolar affective disorder, and non-organic sleep disorders. These findings indicate that late-life depression pharmacotherapy in this cohort was rarely isolated antidepressant prescribing. Instead, it occurred in a broader psychogeriatric context characterized by anxiety, insomnia, cognitive or organic psychiatric morbidity, somatic co-treatment, and repeated service contact. This is clinically plausible, because psychopharmacological treatment in older adults is shaped by altered pharmacokinetics and pharmacodynamics, multimorbidity, central nervous system sensitivity, and the need to balance symptom control against adverse drug events Kratz and Diefenbacher (1)Jacobson (2)Pazan and Wehling (6)Skou et al. (7)Toh et al. (8).

Psychiatric complexity added further explanatory value. In the depressive-episode subgroup, the number of unique psychiatric diagnoses was independently associated with the annual major DDI rate, while anxiety disorders were associated with the annual total potential DDI rate. Other mental disorders due to brain injury, brain dysfunction, or somatic condition were associated with higher annual major DDI rates in the diagnosis-extended model, and bipolar affective disorder showed a non-significant trend in the same direction. These results suggest that diagnostic complexity contributes to cumulative annual DDI burden, even when the regression models include medication load. This is consistent with routine psychogeriatric practice, where anxiety symptoms, sleep complaints, cognitive impairment, affective instability, psychotic symptoms, and somatic morbidity may generate layered prescribing trajectories across time. The distinction between rational and irrational polypharmacy remains important: combination therapy may be clinically justified in complex psychiatric illness, but combinations with overlapping sedative, anticholinergic, cardiovascular, electrolyte, or pharmacokinetic liabilities require periodic reassessment Zigman and Blier (11)Preskorn and Lacey (12)Nakagami et al. (47).

### 4.2 Multidimensional prescribing risk and cumulative vulnerability

Medication load was the most consistent factor associated with DDI burden among patients with depressive episodes. Each additional mean drug per visit was associated with higher odds of any major DDI and with higher annual total potential and major potential DDI rates. This result highlights the expected link between regimen size and interaction burden, emphasizing that in late-life depression, DDI risk extends beyond just antidepressants. It emerges from the cumulative structure of regimens that combine antidepressants, anxiolytics, hypnotics, antipsychotics, mood stabilizers, cognitive enhancers, cardiovascular drugs, diuretics, antiplatelet agents, and other somatic drugs. Prior work has similarly emphasized that polypharmacy, psychiatric illness, and DDI exposure overlap substantially in older and psychiatric populations, while not being perfectly interchangeable constructs Al Zaabi et al. (10)Hefner et al. (17)Tulner et al. (18)Hughes et al. (19). These findings are also consistent with recent geriatric DDI studies showing that potential DDIs are common among older adults and are strongly related to polypharmacy and outpatient prescribing context Bories et al. (48)Liu et al. (49). The central role of medication count is also consistent with recent large-scale outpatient pharmacoepidemiologic evidence. Aarabi et al. reported a substantial prevalence of potential DDIs in national outpatient prescribing data and identified polypharmacy and specific prescribing patterns as major predictors, with psychiatry among the higher-risk prescribing contexts Aarabi et al. (43). Accordingly, medication review should begin with medication count but extend to the overall risk architecture of the regimen.

The diagnosis-specific DDI rankings identify clinically recognizable medication review targets. Because several leading pairs recurred across depressive episode, anxiety disorder, and same-visit depressive and anxiety overlap subgroups, **Table 7** summarizes the 10 unique leading potential DDI pairs from **Table 4**, focusing on their mechanism, potential clinical consequence, and medication review consideration. We present this table to contextualize DrugBank-derived severity labels, not to imply that each potential DDI was clinically harmful or automatically inappropriate. Among patients with depressive episodes, leading moderate DDIs included alprazolam + zolpidem, lorazepam + zolpidem, lorazepam + mirtazapine, and lorazepam + trazodone. The same pattern was particularly visible in the same-visit depressive and anxiety overlap subgroup, where moderate DDI exposure was almost universal at the visit level. These combinations reflect a common therapeutic dilemma in late-life depression: anxiety and insomnia are frequent clinical drivers of prescribing, and benzodiazepines, Z-drugs, sedating antidepressants, and other psychotropics may be used together in routine care. However, in older adults, such combinations can accumulate sedative, cognitive, psychomotor, orthostatic, and fall-related effects even when each individual drug is prescribed at a usual dose. Benzodiazepines and Z-drugs are consistently highlighted as problematic in geriatric practice because of fall risk, cognitive impairment, and other adverse outcomes Capiau et al. (55)Díaz-Gutiérrez et al. (56)Liu et al. (59)Islam et al. (60). Trazodone, although often perceived as a safer hypnotic alternative, has also been associated with fall-related injury in older nursing home residents Bronskill et al. (57). Therefore, moderate DDIs should not be dismissed as clinically unimportant in older patients with depressive episodes; they should prompt review of indication, dose, duration, timing, cumulative sedative burden, and deprescribing opportunities.

**Table 7:** Pharmacological interpretation and medication-review considerations for the 10 unique leading potential DDI pairs.

Potential DDI pair	Likely mechanism	Potential clinical consequence	Medication-review consideration
Atorvastatin + clopidogrel	Potential CYP3A4-mediated interaction affecting clopidogrel activation; evidence suggests possible attenuation of platelet inhibition during concomitant atorvastatin use, although clinical significance remains context-dependent Knox et al. (45)Lau et al. (50)Ferreira et al. (51).	Possible attenuation of clopidogrel antiplatelet efficacy, with lower platelet inhibition reported during concomitant atorvastatin use; clinical significance remains context-dependent.	Review cardiovascular indication, thrombotic risk, antiplatelet response, and statin choice; consider monitoring or a non-CYP3A4 statin strategy when clinically indicated.
Indapamide + trazodone	Increased QTc prolongation risk; clinically, this may reflect combined electrophysiological and electrolyte vulnerability Pacher and Kecskemeti (52)Knox et al. (45)Raja et al. (53).	Increased arrhythmia vulnerability, especially in patients with hypokalemia, other electrolyte abnormalities, baseline QT/TdP risk factors, or concomitant QT-prolonging drugs.	Monitor electrolytes and ECG in high-risk patients; review orthostatic symptoms, blood pressure, and other QT/TdP risk factors.
Alprazolam + carbamazepine	Primarily CYP3A4 induction: carbamazepine may increase alprazolam metabolism and reduce alprazolam effect; DrugBank also flags possible altered carbamazepine metabolism Fuhr et al. (54)Knox et al. (45).	Reduced alprazolam anxiolytic and sedative efficacy, possible loss of symptom control, and potential reflex dose escalation; additive CNS effects may also occur.	Monitor anxiety and insomnia control and alprazolam efficacy; avoid reflex dose escalation without reassessing the interaction; monitor sedation and carbamazepine tolerability.
Alprazolam + zolpidem	Additive CNS depressant and psychomotor impairing effects American Geriatrics Society (31)Capiau et al. (55)D´ıaz-Gutierrez´ et al. (56).	Excessive sedation, impaired attention, psychomotor slowing, cognitive impairment, falls, and fall-related injury.	Avoid routine sedative duplication when possible; use the lowest effective doses and shortest duration; reassess insomnia and anxiety treatment, and monitor sedation, cognition, and falls risk.
Lorazepam + zolpidem	Additive CNS depressant and psychomotor impairing effects American Geriatrics Society (31)Capiau et al. (55)D´ıaz-Gutierrez´ et al. (56).	Increased sedation, next-day impairment, confusion, psychomotor impairment, falls, and fall-related injury.	Reassess need for benzodiazepine plus Z-drug therapy; consider dose/timing adjustment, tapering, or non-pharmacological insomnia strategies; counsel regarding falls and impairment.
Lorazepam + mirtazapine	Additive sedative and CNS depressant effects Knox et al. (45).	Daytime somnolence, dizziness, impaire concentration, impaired balance, and risk of falls.	Review cumulative sedative burden, dose, and timing of administration; monitor cognition, gait instability, daytime somnolence, and risk of falls.
Lorazepam + trazodone	Additive CNS depressant effects, with trazodone also associated with dizziness and orthostatic hypotension; low-dose trazodone and benzodiazepines can cause fall-related injuries in older adults Bronskill et al. (57), 45.	Sedation, dizziness, orthostasis, falls, and fall-related injury.	Reassess use of trazodone as a hypnotic/sedating antidepressant in combination with a benzodiazepine; monitor orthostatic hypotension, sedation, cognition, and risk of falls.
Atorvastatin + carbamazepine	Carbamazepine is a strong enzyme inducer and may reduce atorvastatin exposure, particularly through CYP3A4-related pathways 54Bullman et al. (58).	Reduced lipid-lowering efficacy.	Monitor lipid control response; review cardiovascular indication; consider dose adjustment or a statin less dependent on CYP3A4, when clinically appropriate.
Alprazolam + trazodone	Additive CNS depressant and sedative effects, with possible orthostatic burden Knox et al. (45)Capiau et al. (55).	Sedation, dizziness, impaired psychomotor performance, orthostatic hypotension, and risk of falls.	Reassess combined anxiolytic and sedating-antidepressant use; consider dose and timing adjustment and monitor excessive sedation and risk of falls.
Bisoprolol + spironolactone	Pharmacodynamic cardiovascular and electrolyte interaction; increased hyperkalemia risk during coadministration Knox et al. (45).	Hyperkalemia, especially with renal impairment, diabetes, frailty, or other potassium-raising drugs; possible dizziness, hypotension, weakness, or bradycardia.	Monitor serum potassium, renal function, blood pressure, and heart rate; review concomitant ACE inhibitors/ARBs, potassium supplements, NSAIDs, or other potassium-raising drugs.

CNS, central nervous system; DDI, drug-drug interaction; QT/TdP, QT prolongation/torsades de pointes; ACE, Angiotensin-Converting enzyme; ARBs, Angiotensin II receptor blockers; NSAIDs, Nonsteroidal anti-inflammatory drugs.

The table summarizes the unique potential DDI pairs listed in **Table 4**. Mechanisms and medication review considerations are intended to contextualize database-derived severity labels, not to imply that every potential DDI is clinically harmful or inappropriate.

The major DDI profile was more heterogeneous and included cardiovascular, antiepileptic, antidepressant, and psychotropic combinations. Atorvastatin + clopidogrel was the leading major DDI pair across depressive-episode, anxiety disorder, and depressive/anxiety overlap analyses. This signal should not be interpreted as indicating that such combinations are uniformly inappropriate. Instead, the main concern is possible attenuation of clopidogrel antiplatelet efficacy, not a direct toxicity; lower platelet inhibition has been reported during concomitant clopidogrel and atorvastatin use, although the clinical significance remains context-dependent Ferreira et al. (51). This signal, therefore, reflects the need for contextual review when psychiatric treatment intersects with cardiovascular prevention and related cardiovascular comorbidities. Depression and anxiety may coexist with cardiovascular risk, and treatment regimens in older adults frequently span several specialties Pazan and Wehling (6)Skou et al. (7)Civieri et al. (61). Other major DDI pairs involving carbamazepine were also clinically plausible because carbamazepine is a strong enzyme inducer with substantial pharmacokinetic interaction potential U.S. Food and Drug Administration (16)Fuhr et al. (54). For atorvastatin + carbamazepine, the relevant concern is reduced lipid-lowering efficacy Bullman et al. (58), whereas for alprazolam + carbamazepine, pharmacokinetic evidence supports reduced alprazolam exposure through carbamazepine-mediated CYP3A4 induction Spina and Perucca (62). These findings support cross-specialty medication review: DDI prevention in late-life depression should include both psychotropic combinations and interactions between psychotropics and somatic drugs.

A central message of our study is that prescribing risk in older adults with depressive episodes is best conceptualized as cumulative vulnerability rather than as a simple count of interacting pairs. In addition to DDI counts, the higher-burden prescribing risk profile showed higher anticholinergic burden, QT/TdP risk drug burden, Beers burden, SIADH risk drug exposure, and peak prescribing intensity. These dimensions are clinically relevant in late-life depression because they may converge on common geriatric outcomes such as confusion, falls, cognitive worsening, syncope, arrhythmia, hyponatremia, hospitalization, and reduced functional reserve. The Beers-related component of the prescribing risk profile is supported by recent outpatient geriatric evidence from more than 327,000 older outpatients and 1.27 million prescription medications, showing that 21% of prescriptions included at least one PIM according to the 2023 Beers Criteria, while polypharmacy was present in 36.95% of prescriptions Gholamnezhad et al. (42). This supports interpreting DDI burden together with geriatric medication risk domains and not as an isolated pairwise interaction count. Anticholinergic burden is relevant to cognitive and neuropsychiatric vulnerability in older adults Boustani et al. (20)López-Álvarez et al. (22)Taylor-Rowan et al. (23). QT/TdP risk burden is clinically important when psychotropics are combined with other QT-prolonging drugs or used in patients with cardiovascular vulnerability Beach et al. (25)Meid et al. (26)Ramasubbu et al. (27). SIADH and hyponatremia are also relevant because antidepressants, antipsychotics, antiepileptics, and diuretics may contribute to electrolyte disturbances that can mimic or worsen cognitive impairment, gait instability, or falls Cuesta and Thompson (29)Warren et al. (30). PIM frameworks such as Beers, STOPP/START, and PRISCUS were developed precisely because older adults are vulnerable to cumulative, context-dependent prescribing hazards that are not captured by drug count alone O’Mahony et al. (5)American Geriatrics Society (31)Thürmann et al. (32).

The profile analysis supports this multidimensional interpretation. The retained two-profile solution separated a lower-burden profile from a higher-burden profile along a dominant prescribing risk axis, with PC1 explaining nearly half of the variance. The higher-burden profile was characterized by approximately double the mean drug exposure per visit and nearly four times the mean total potential DDI burden per visit compared with the lower-burden profile, together with higher major, moderate, and minor DDI burden and higher geriatric medication risk scores. Importantly, depressive-episode patients were represented in both profiles: 35.5% belonged to the higher-burden profile, compared with 33.9% of patients in the same-visit depressive and anxiety overlap subgroup. Thus, the profile should not be interpreted as a depression-specific cluster; it identifies a prescribing risk gradient within the psychogeriatric care setting in which late-life depressive episodes are treated.

Exploratory sex-stratified analyses suggested that men with depressive episodes had higher medication load and higher total DDI burden than women, despite similar psychiatric diagnostic burden. This pattern should be interpreted cautiously because the analysis was descriptive and involved a smaller male subgroup. It also differs from several population-based studies in which women showed higher psychotropic medication use, polypharmacy, PIM exposure, or DDI risk Boyd et al. (63)Cebrino and Portero de la Cruz (64)Alwhaibi and Balkhi (65)Correia et al. (66). One possible explanation is that our analysis focused on older psychogeriatric patients with depressive episodes, in whom higher DDI burden among men may reflect greater somatic co-treatment, cardiovascular medication exposure, or different prescribing trajectories rather than greater psychiatric diagnostic burden. These findings therefore support sex-stratified medication review as an exploratory signal, but they should not be interpreted as evidence of a generalizable male-specific DDI mechanism.

### 4.3 Clinical implementation framework

**Figure 5:**
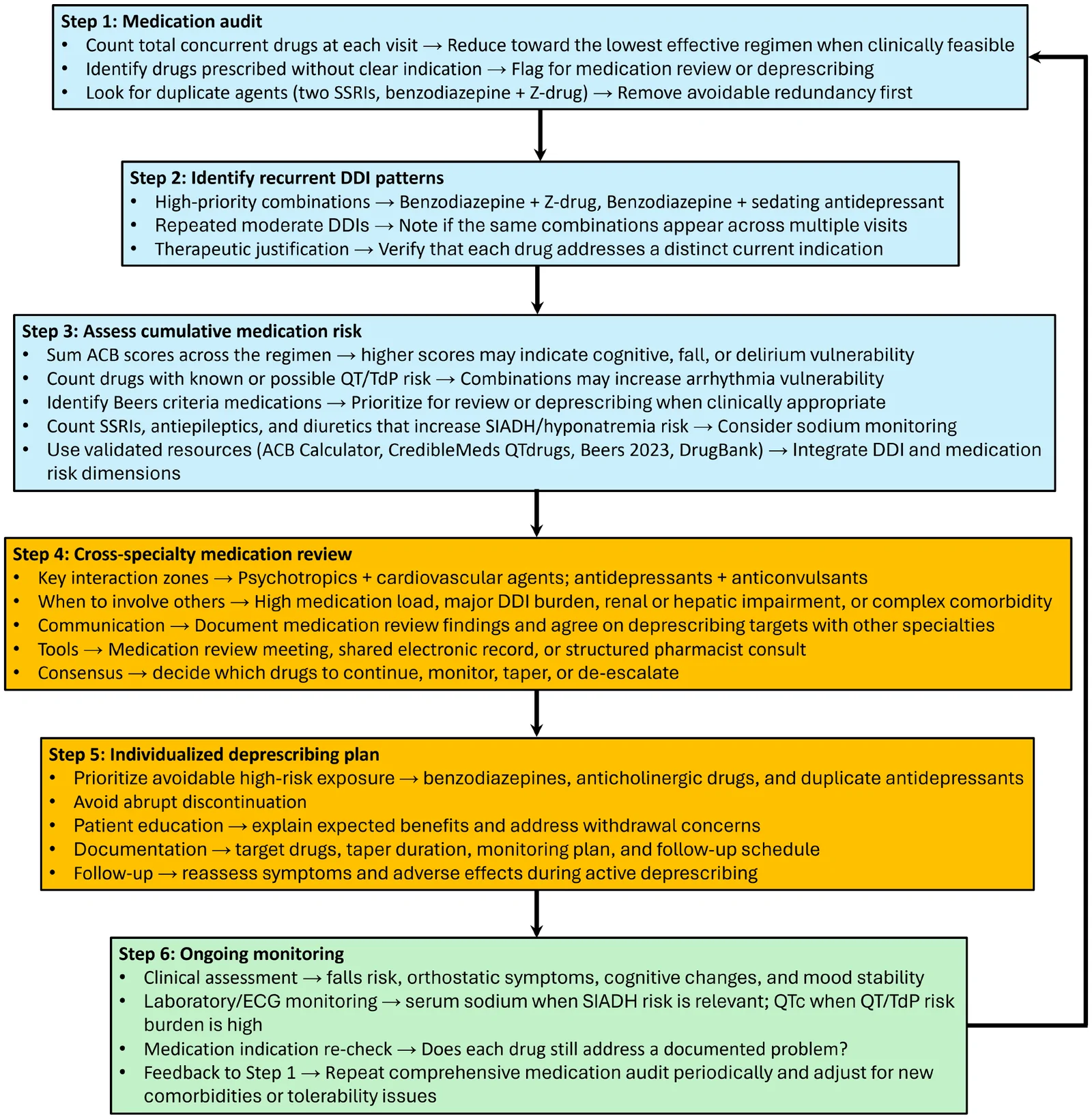
Proposed structured medication review framework for late-life depression. The framework summarizes a practical six-step approach derived from the study findings. It is organized into three phases: assessment (Steps 1–3), including medication-load review, recurrent DDI-pattern screening, and cumulative medication risk assessment; action (Steps 4–5), including cross-specialty medication review and individualized deprescribing; and monitoring (Step 6), including surveillance for adverse outcomes, laboratory or ECG monitoring when clinically indicated, and periodic reassessment of medication need. (ACB, Anticholinergic Cognitive Burden; DDI, drug-drug interaction; ECG, electrocardiogram; QTc, corrected QT interval; QT/TdP, QT prolongation/torsades de pointes; SIADH, syndrome of inappropriate antidiuretic hormone secretion).

From a clinical implementation perspective, these findings support a structured, regimen-level medication review approach for older adults with depressive episodes (**Figure 5**). The proposed framework begins with medication load assessment, because drug count was the most reproducible determinant of DDI burden, but then moves beyond drug count to identify recurrent DDI patterns and cumulative geriatric medication risk burden. Each drug should be linked to a current indication, and drugs without a clear ongoing benefit should be flagged for medication review. Clinicians must pay attention to therapeutic duplication, including drugs prescribed for the same diagnosis with similar pharmacological activity; such combinations may increase DDI burden without adding meaningful benefit.

The second step involves reviewing DDI patterns. Major DDIs should lead to a contextual reassessment of the interacting drugs, with particular attention to medications with broad interaction potential, such as strong enzyme inducers like carbamazepine. Moderate DDIs should not be dismissed in older adults, especially when they recur across multiple visits or involve sedative, anxiolytic, hypnotic, or antidepressant combinations. Instead, their clinical relevance should be assessed at the patient level, considering age, frailty, cognitive status, risk of falls, cardiovascular vulnerability, electrolyte risk, and availability of safer alternatives. Pharmacodynamic DDIs also require interpretation in context: some combinations may reflect intentional augmentation strategies or attempts to manage adverse effects, but these should be periodically reassessed to ensure that each drug still has a justified role in the regimen.

The third assessment step quantifies cumulative medication risk dimensions using validated frameworks, including anticholinergic burden, QT/TdP risk burden, Beers criteria exposure, and SIADH-related risk.

The framework then moves to the action phase, where the clinician collaborates with cross-specialty partners to understand psychiatric and somatic DDIs. This is particularly relevant when psychotropic treatment intersects with cardiovascular, neurological, or internal medicine co-treatment. In the deprescribing step, clinicians should develop an individualized plan with gradual dose reductions, prioritizing avoidable sedative, anticholinergic, duplicate, or poorly justified combinations while preserving clinically necessary therapy, consistent with recent deprescribing guidance for older adults with polypharmacy Hung et al. (67).

The third phase is ongoing monitoring. Clinicians should reassess falls risk, orthostatic symptoms, cognitive status, mood stability, medication tolerability, and whether each drug still addresses a documented clinical problem. Laboratory or ECG monitoring may be appropriate for specific risks, such as serum sodium monitoring for SIADH-related risk or QTc for high QT/TdP risk. This phase should inform a periodic comprehensive medication audit, highlighting that medication review in late-life depression is a continuous process, not a one-time intervention. The goal is not to eliminate all polypharmacy, but to distinguish clinically justified combination therapy from avoidable cumulative prescribing risk Zigman and Blier (11)Preskorn and Lacey (12)O’Donnell and Ibrahim (14)Linsky et al. (15).

Recent evidence also supports the feasibility of electronic prescribing and outpatient prescription surveillance as a medication safety infrastructure. Electronic alerts based on the Beers criteria may help reduce potentially inappropriate prescribing in older adults Aminzade et al. (68), while standardized outpatient prescribing surveillance can support monitoring and rational prescribing interventions at scale Nasehi et al. (69). Therefore, the framework proposed in **Figure 5** should be interpreted not as a validated intervention, but as a clinically oriented structure that could be implemented and tested within electronic prescribing or pharmacist-supported medication review systems. The relevance of structured medication review in Romanian outpatient care is also supported by local STOPP/START evidence. Buda et al. found PIMs and prescribing omissions in rural elderly patients using STOPP/START version 2 criteria, emphasizing the need for interdisciplinary prescription review Buda et al. (70). More recent Western Romanian outpatient evidence emphasized under-prescription detected with START criteria, reinforcing that medication safety review should consider not only potentially harmful combinations, but also omissions and broader pharmacotherapy optimization Baneu et al. (71).

These findings also have implications for DDI alert interpretation. Major DDIs remain important, but moderate DDIs deserve greater attention in older adults with depressive episodes because they can accumulate across repeated visits and combine with age-related vulnerability. Standard alert systems may underemphasize this cumulative pattern when each pair is classified as moderate. In addition, DDI databases and electronic interaction checkers may differ in how interactions are reported, categorized, and prioritized, which reinforces the need for clinical contextualization rather than automatic acceptance of severity labels Suciu et al. (72)Udrescu et al. (73). In late-life depression, repeated moderate interactions involving sedatives, hypnotics, anxiolytics, antidepressants, and antiepileptics may be clinically important, particularly when the patient also has cognitive impairment, vascular disease, falls risk, hyponatremia risk, or anticholinergic exposure. Recent multidimensional approaches, such as the PsychoPharm Aggregated Risk Score (PARS), further support the integration of DDI burden with anticholinergic, QT/TdP, and other psychopharmacological risk dimensions when flagging high-risk regimens Goldiş et al. (74). Unlike PARS, which was developed as an aggregated risk score, the present study used unsupervised exploratory profiling to characterize prescribing risk patterns in older adults with depressive episodes. Medication review should therefore prioritize the overall risk architecture of the regimen instead of focusing on the highest-severity DDI pairs.

### 4.4 Limitations and future directions

We must acknowledge several limitations. (1) The study was retrospective and observational, so findings describe potential DDIs and prescribing risk profiles and not confirmed adverse drug events. (2) DDI classification was database-driven and did not account for dose, duration, route, frequency, *pro re nata* status, indication, renal or hepatic function, frailty, pharmacogenetic variation, adherence, or clinician monitoring. (3) In the present study, falls, delirium, hospitalization, arrhythmia, serum sodium, or treatment response were not measured outcomes; therefore, the identified combinations should be interpreted as medication review signals and not as observed adverse drug events. (4) Profile analysis was exploratory and should be validated in independent cohorts before clinical implementation. (5) The single-center design limits its generalizability to other healthcare systems, formularies, and prescribing cultures. (6) We analyzed only drugs recorded in the structured medication list at each visit and did not validate against pharmacy dispensing records; therefore, we cannot confirm actual medication adherence and any over-the-counter or self-medications.

Despite these limitations, this study opens several avenues for future research. (1) Prospective validation of the six-step medication review framework in real-world clinical settings would establish whether it improves medication safety outcomes compared to usual care. (2) Implementation science approaches should explore barriers and facilitators for adopting the framework in diverse healthcare settings, which would guide scalability. (3) Integration of the framework into electronic health record systems with automated cumulative risk scoring (combining DDI, anticholinergic, QT/TdP, Beers, and SIADH dimensions) could reduce clinician burden and improve adherence to evidence-based medication review without requiring manual risk calculation. (4) Assessing whether deprescribing interventions based on this framework enhance outcomes and quality of life for older adults would determine clinical effectiveness. (5) International validation of these findings in healthcare systems with different formularies, prescribing cultures, and access to psychotropic medications would determine generalizability beyond the Romanian setting.

## 5 Conclusion

Older adults with depressive episodes receiving psychogeriatric care experienced substantial DDI burden within a diagnostically complex and pharmacologically intensive care context. Medication load was the primary driver of interaction burden, but psychiatric diagnostic complexity contributed to annual major DDI rates. The frequent overlap with anxiety disorders, the recurrence of sedative-hypnotic and anxiolytic-antidepressant moderate DDIs, the presence of carbamazepine-related interaction pathways and cardiovascular co-treatment, and the accumulation of geriatric medication risk scores all suggest that safer pharmacological management of late-life depression requires review of the entire regimen across psychiatric and somatic care. The prescribing risk profiles further show that a clinically important subgroup of patients with depressive episodes belongs to a higher-burden profile characterized by polypharmacy, DDI accumulation, and elevated geriatric medication risk exposure. These findings support structured medication review, careful interpretation of repeated moderate DDIs, and individualized deprescribing strategies as core components of safer psychopharmacological management of depression in older adults.

## Data Availability

The data analyzed in this study is subject to the following licenses/restrictions: The datasets for this article are not publicly available due to concerns regarding patient anonymity. Requests to access these datasets should be directed to udrescu.lucretia@umft.ro.
