# Supplementary material for: Exploratory prescribing risk profiles and drug drug interaction burden in older adults with depressive episodes
Source: Front Psychiatry. 2026 Aug 21;17:1915707. doi: 10.3389/fpsyt.2026.1915707 (PMC13541716; doi:10.3389/fpsyt.2026.1915707)
Supplement: Supplementary file 1 [file DataSheet1.pdf]

Supplementary Material

Supplementary Table S1. Psychiatric diagnosis codes from the Romanian 999-code disease list used in the present analysis.

The table lists the psychiatric diagnosis-code subset relevant to the present psychogeriatric analysis. English labels are provided for interpretability. Code 321 defined the primary depressive-episode subgroup, code 325 defined the anxiety-disorder subgroup, and same-visit depressive/anxiety overlap was defined by the co-occurrence of codes 321 and 325 in the same visit record.

Supplementary Table S2. Sex-stratified characteristics of patients with depressive episodes.

Values are presented as n (%), mean (SD), or mean (SD); median [IQR], as appropriate. Analyses are restricted to patients with at least one depressive episode diagnosis during 2023. p-values are exploratory and were calculated using Mann–Whitney U tests for continuous variables and chi-square or Fisher exact tests for categorical variables, as appropriate; they were not adjusted for multiple comparisons. Other organic/somatic mental disorders refers to other mental disorders due to brain injury, brain dysfunction, or somatic condition. True DDI pairs are the sum of major, moderate, and minor DDI pairs.

Supplementary Table S3. Full patient-level rankings of major and moderate drug-drug interaction pairs in depression- and anxiety-related analytic subgroups.

Patient-level prevalence was calculated as the number of unique patients in each subgroup who had the specified drug-drug interaction pair at least once during 2023. The same-visit depressive/anxiety overlap subgroup was defined as patients with at least one visit in which depressive episode code 321 and anxiety disorder code 325 were recorded together.

Supplementary Table S1. Psychiatric diagnosis codes from the Romanian 999-code disease list used in the present analysis.

Code	English diagnosis label	Code	English diagnosis label
299	Vascular dementia	328	Dissociative [conversion] disorders
300	Dementia, unspecified	329	Somatoform disorders
301	Organic amnesic syndrome, not due to alcohol or psychoactive substances	330	Other neurotic disorders
302	Delirium, not induced by alcohol or other psychoactive substances	331	Eating disorders
303	Other mental disorders due to brain damage	332	Non-organic sleep disorders
304	Personality and behavioral disorders due to disease	333	Sexual dysfunction not due to a disorder or disease
305	Organic or symptomatic mental disorders, unspecified	334	Mental and behavioral disorders associated with the puerperium, unspecified
306	Mental and behavioral disorders due to alcohol use	335	Psychological and behavioral factors associated with diseases and disorders
307	Mental and behavioral disorders due to opiate use and derivatives	336	Abuse of substances not leading to dependence
308	Mental and behavioral disorders due to sedative or hypnotic use	337	Unspecified behavioral syndrome associated with physiological disturbances
309	Mental and behavioral disorders due to stimulating substance use	338	Specific personality disorders
310	Mental and behavioral disorders due to tobacco use	339	Gender identity disorders and sexual preference disorders
311	Mental and behavioral disorders due to volatile solvent use	340	Psychological and behavioral problems associated with sexual development
312	Schizophrenia	341	Other personality and adult behavior disorders
313	Schizotypal disorder	342	Mild mental retardation
314	Persistent delusional disorders	343	Moderate mental retardation
315	Acute and transient psychotic disorders	344	Severe mental retardation
316	Induced delusional disorder	345	Profound mental retardation
317	Schizoaffective disorders	346	Other unspecified forms of mental retardation
318	Other non-organic psychotic disorders	347	Specific developmental speech and language disorders
319	Manic episode	348	Specific developmental motor disorders
320	Bipolar affective disorder	349	Other psychological development disorders, unspecified
321	Depressive episode	350	Hyperkinetic disorders
322	Disorders of mood (affective disorders)	351	Conduct disorders
323	Other mood (affective) disorders	352	Emotional disorders with onset specific to childhood
324	Affective mood disorders, unspecified	353	Social functioning disorders with onset specific to childhood
325	Anxiety disorders	354	Other behavioral and emotional disorders in childhood
326	Obsessive-compulsive disorder	355	Mental disorder, unspecified
327	Reaction to severe stress and adjustment disorders		

Source: Romanian Ministry of Health, Romanian 999-code disease list aligned with the International Classification of Diseases, 10th Revision. The table includes only the subset of psychiatric codes relevant to the present analysis.

Supplementary Table S2. Sex-stratified characteristics of patients with depressive episodes.

Characteristic	Women (n=182)	Men (n=60)	p-value
**Demographic and diagnostic characteristics**			
Age at first visit, years	75.93 (7.88); 75 [69–82]	75.98 (7.92); 75 [70–81]	0.957
Visits per patient	4.12 (3.50); 3 [1–6]	4.55 (3.60); 3 [2–6]	0.286
Unique psychiatric diagnoses	2.79 (1.25); 2 [2–4]	2.90 (1.41); 3 [2–4]	0.653
Patients with ≥2 unique psychiatric diagnoses, n (%)	165 (90.7%)	53 (88.3%)	0.784
Patients with ≥3 unique psychiatric diagnoses, n (%)	88 (48.4%)	32 (53.3%)	0.603
**Psychiatric co-diagnoses**			
Anxiety disorders, n (%)	144 (79.1%)	49 (81.7%)	0.810
Same-visit depressive/anxiety overlap, n (%)	136 (74.7%)	41 (68.3%)	0.423
Vascular dementia, n (%)	40 (22.0%)	13 (21.7%)	1.000
Other organic/somatic mental disorders, n (%)	39 (21.4%)	21 (35.0%)	0.053
Acute and transient psychotic disorders, n (%)	30 (16.5%)	8 (13.3%)	0.706
Bipolar affective disorder, n (%)	16 (8.8%)	5 (8.3%)	1.000
Non-organic sleep disorders, n (%)	17 (9.3%)	3 (5.0%)	0.419
**Medication and DDI burden**			
Mean drugs per visit	5.87 (2.75)	6.76 (2.88)	0.029
Annual true DDI pairs	45.19 (58.24); 23 [6–62]	66.92 (81.49); 31 [14–76]	0.031
Annual major DDI pairs	4.84 (8.98); 1 [0–5]	6.30 (10.52); 2 [0–7]	0.066
Mean true DDI pairs per visit	10.10 (8.73)	13.67 (10.08)	0.013
Mean major DDI pairs per visit	0.96 (1.43)	1.26 (1.64)	0.079
Patients with ≥1 major DDI, n (%)	94 (51.6%)	40 (66.7%)	0.060
Patients with ≥1 moderate DDI, n (%)	172 (94.5%)	58 (96.7%)	0.735
Patients with ≥1 moderate-or-major DDI, n (%)	175 (96.2%)	58 (96.7%)	1.000
**Medication risk burden**			
Mean ACB burden per visit	1.90 (1.57)	2.09 (1.82)	0.621
Mean QT/TdP risk burden per visit	2.05 (1.44)	2.13 (1.20)	0.386
Mean Beers burden per visit	5.66 (2.88)	5.54 (3.27)	0.807
Mean SIADH risk drugs per visit	0.95 (0.77)	0.92 (0.89)	0.805
**Prescribing risk phenotype**			
Higher-burden prescribing-risk phenotype, n (%)	59 (32.4%)	27 (45.0%)	0.107

Abbreviations: ACB, Anticholinergic Cognitive Burden; DDI, drug-drug interaction; IQR, interquartile range; QT/TdP, QT prolongation/torsades de pointes; SD, standard deviation; SIADH, syndrome of inappropriate antidiuretic hormone secretion.

Supplementary Table S3. Full patient-level rankings of major and moderate drug-drug interaction pairs in depression- and anxiety-related analytic subgroups.

Supplementary Table S3A. Patients with depressive episodes during 2023 (n=242 patients).

Severity	Rank	DDI pair	Patients, n (%)
Major	1	Atorvastatin + Clopidogrel	16 (6.6%)
Major	2	Indapamide + Trazodone	13 (5.4%)
Major	3	Alprazolam + Carbamazepine	12 (5.0%)
Major	4	Atorvastatin + Carbamazepine	10 (4.1%)
Major	5	Bisoprolol + Spironolactone	10 (4.1%)
Major	6	Carbamazepine + Zopiclone	9 (3.7%)
Major	7	Carbamazepine + Nebivolol	8 (3.3%)
Major	8	Nebivolol + Spironolactone	8 (3.3%)
Major	9	Quetiapine + Trazodone	8 (3.3%)
Major	10	Amlodipine + Carbamazepine	7 (2.9%)
Moderate	1	Alprazolam + Zolpidem	23 (9.5%)
Moderate	2	Lorazepam + Zolpidem	23 (9.5%)
Moderate	3	Lorazepam + Mirtazapine	19 (7.9%)
Moderate	4	Lorazepam + Trazodone	19 (7.9%)
Moderate	5	Alprazolam + Tianeptine	17 (7.0%)
Moderate	6	Alprazolam + Trazodone	17 (7.0%)
Moderate	7	Bromazepam + Mirtazapine	15 (6.2%)
Moderate	8	Carbamazepine + Memantine	15 (6.2%)
Moderate	9	Bisoprolol + Indapamide	14 (5.8%)
Moderate	10	Atorvastatin + Mirtazapine	13 (5.4%)

Supplementary Table S3B. Patients with anxiety disorders during 2023 (n=232 patients).

Severity	Rank	DDI pair	Patients, n (%)
Major	1	Atorvastatin + Clopidogrel	15 (6.5%)
Major	2	Alprazolam + Carbamazepine	13 (5.6%)
Major	3	Atorvastatin + Carbamazepine	12 (5.2%)
Major	4	Bisoprolol + Spironolactone	11 (4.7%)
Major	5	Indapamide + Trazodone	11 (4.7%)
Major	6	Amlodipine + Carbamazepine	8 (3.4%)
Major	7	Carbamazepine + Zopiclone	8 (3.4%)
Major	8	Quetiapine + Trazodone	8 (3.4%)
Major	9	Bromazepam + Carbamazepine	7 (3.0%)
Major	10	Candesartan + Spironolactone	7 (3.0%)
Moderate	1	Lorazepam + Zolpidem	28 (12.1%)
Moderate	2	Alprazolam + Zolpidem	26 (11.2%)
Moderate	3	Alprazolam + Trazodone	19 (8.2%)
Moderate	4	Lorazepam + Mirtazapine	19 (8.2%)
Moderate	5	Lorazepam + Trazodone	19 (8.2%)
Moderate	6	Carbamazepine + Memantine	17 (7.3%)
Moderate	7	Alprazolam + Tianeptine	16 (6.9%)
Moderate	8	Alprazolam + Escitalopram	15 (6.5%)
Moderate	9	Alprazolam + Zopiclone	15 (6.5%)
Moderate	10	Bromazepam + Mirtazapine	14 (6.0%)

Supplementary Table S3C. Patients with at least one same-visit depressive/anxiety overlap during 2023 (n=177 patients).

Severity	Rank	DDI pair	Patients, n (%)
Major	1	Atorvastatin + Clopidogrel	10 (5.6%)
Major	2	Indapamide + Trazodone	8 (4.5%)
Major	3	Atorvastatin + Carbamazepine	7 (4.0%)
Major	4	Bisoprolol + Spironolactone	7 (4.0%)
Major	5	Bromazepam + Carbamazepine	5 (2.8%)
Major	6	Carbamazepine + Zopiclone	5 (2.8%)
Major	7	Quetiapine + Trazodone	5 (2.8%)
Major	8	Alprazolam + Carbamazepine	4 (2.3%)
Major	9	Amiodarone + Apixaban	4 (2.3%)
Major	10	Amlodipine + Carbamazepine	4 (2.3%)
Moderate	1	Lorazepam + Trazodone	19 (10.7%)
Moderate	2	Alprazolam + Zolpidem	18 (10.2%)
Moderate	3	Lorazepam + Mirtazapine	18 (10.2%)
Moderate	4	Lorazepam + Zolpidem	16 (9.0%)
Moderate	5	Alprazolam + Tianeptine	15 (8.5%)
Moderate	6	Alprazolam + Trazodone	15 (8.5%)
Moderate	7	Bromazepam + Mirtazapine	14 (7.9%)
Moderate	8	Atorvastatin + Mirtazapine	13 (7.3%)
Moderate	9	Bromazepam + Zolpidem	13 (7.3%)
Moderate	10	Bromazepam + Trazodone	12 (6.8%)
